# What are women’s experiences of gestational breast cancer, and how do they describe their interactions with the healthcare system? An exploratory study

**DOI:** 10.1177/17455057261435753

**Published:** 2026-03-29

**Authors:** Sara Hurren, Marie McAuliffe, Karen Yates, Tracey Ahern, Cate Nagle

**Affiliations:** 1 College of Healthcare Sciences, James Cook University, Cairns, QLD, Australia; 2 College of Healthcare Sciences, James Cook University, The Townsville Hospital and Health Service, Townsville City, QLD, Australia; 3 Centre for Quality and Safety Research-Barwon Health Partnership, Deakin University, Geelong, VIC, Australia

**Keywords:** breast-neoplasms, pregnancy, women’s health, reproduction, delivery in healthcare

## Abstract

**Background:**

Gestational breast cancer (GBC) is defined as a breast cancer diagnosis during pregnancy or within 12 months post-partum. The incidence of GBC varies between 1:1000 and 1:3000 pregnancies. Breast cancer is the most frequent malignant tumour in women and the leading cause of cancer-related female mortality worldwide.

**Objectives:**

The main objective of this study was to better understand the experiences of women diagnosed with GBC and their interactions with the healthcare system. By exploring their perspectives, the study aimed to contribute to further research and knowledge to improve care and outcomes for these women.

**Design:**

A qualitative exploratory study.

**Methods:**

The study involved one-on-one semi-structured interviews conducted in Australia between November 2021 and June 2022. Participants were women diagnosed with GBC. Interviews were recorded digitally, transcribed verbatim, and analysed thematically following Braun and Clarke’s (2006) six steps.

**Results:**

Six women diagnosed with GBC participated in the study. Analysis determined three central themes. First: *My happiness was stolen*. Upon receiving a GBC diagnosis, women described feeling terrified, overwhelmed, concerned for their baby, distressed, yet feeling fortunate at the same time. Second: *It really knocked me around*. Women expressed their unique circumstances and difficulties in comprehending having a cancer diagnosis while being pregnant, the different treatment modalities, limited information on surgical and fertility preservation options, and being unprepared for the side effects. Third: *I wanted control*; the importance of being able to take charge of their circumstances and decision-making was important to these women, especially as options were frequently restricted due to the urgency to commence treatment.

**Conclusion:**

The findings provide an understanding of the unique challenges of women diagnosed with GBC. Empowering women through personalised knowledge about their disease, understanding their needs, discussing surgical options, addressing fertility preservation, and providing psychological support is essential.

## Plain language summary

This study interviewed six women to learn about their experiences of being diagnosed with gestational breast cancer (GBC) and their interactions with the healthcare system.

**Why Was the Study Done?** There is limited information available about the experiences of women with GBC. As more women are delaying pregnancy until later in life, those aged 35 years and older are at a higher risk of developing GBC. The study provided a platform for deep, reflective conversations with women who had experienced GBC in Australia, allowing researchers to identify potential improvements in clinical practice and patient care, ultimately supporting these women within the community and healthcare system.

**What Did the Researchers Do?** A qualitative exploratory design was used to explore the experiences, perceptions, and insights of women diagnosed with GBC. The researchers collected qualitative data using semi-structured interviews with six women who had experienced GBC. The interviews were transcribed verbatim and analysed using thematic analysis based on Braun and Clarkes’ (2006) six steps. Thematic analysis is a qualitative research method well-suited for explorative qualitative research, where the goal is to uncover and interpret patterns or themes within the interview transcripts.

**What Did the Researchers Find?** The study identified three main themes and six subthemes that highlight the specific challenges women face due to their age, timing of diagnosis, life stage, and emotional responses to their diagnosis. Participants reported difficulties in understanding their diagnosis, a lack of information, managing treatment side effects, and the complexities of returning home to care for their children.

**What Do the Findings Mean?** The findings highlight the need for healthcare services to recognise the importance of psychological care for women with pregnancy and breast cancer. Healthcare organisations can implement effective communication strategies for each treatment modality plan ensuring women have the information needed to make informed decisions about their health and that of their unborn or newborn child.

## Introduction

Breast cancer is the most frequent cancer among women in the world and the most common cause of cancer death among women.^1–3^ Gestational breast cancer (GBC) is the second most common type of cancer diagnosed in pregnant women, defined as breast cancer detected during pregnancy or within 12 months after giving birth.^1,4–6^ The incidence of GBC is rare, with estimates of 1:1000 and 1:3000 pregnancies.^7^ Women aged 35 years or greater are at increased risk of GBC.^8^

The incidence of GBC globally is reportedly rising, and is thought to be because of increased maternal age, greater screening awareness, breast self-examination, and improved diagnostic and treatment procedures available.^9,10^ Early detection through screening and breast self-examination remains crucial. However, women with GBC, doctors, nurses, and midwives face difficulties in identifying breast cancer symptoms because of similarities in breast changes during pregnancy.^9^

Breast cancer symptoms may include a lump or thickening in the breast, changes to the breast size, shape, or appearance, such as dimpling and redness, changes in nipple appearance, and secretion of abnormal or bloody fluid from the nipple.^11^ Differentiating between breast changes during pregnancy and changes that may indicate a cancer diagnosis remains a challenge. A study by Al-Amri et al.^12^ attributes delays in diagnosing women with GBC to several factors, including uncertainty among healthcare professionals (HCPs), self-delay by women, and healthcare system delays such as processing referrals. These delays may contribute to poorer outcomes for the woman.^13^

A diagnosis of GBC represents a challenging situation for the woman, her family, and the multidisciplinary team because pregnancy adds to the complexity of planning treatment.^7^ While surgery and chemotherapy are safe to administer during the second and third trimester of pregnancy, endocrine and targeted therapies are often not recommended throughout the entire pregnancy.^7^ Women with GBC are often unprepared for the immediate decision-making responsibilities about the health and well-being of their unborn child/children and themselves.^10^ Women diagnosed with GBC often face heightened psychological distress when considering available treatment options.^14^ The added challenge of receiving treatment advice from various HCPs can also make this decision-making process more difficult.^15,16^

Another complexity is in navigating care through different healthcare systems and services. In Australia, the healthcare system comprises a publicly funded system called Medicare, along with a private system.^17^ Medicare has been Australia’s universal healthcare scheme since 1984 and has three major parts: medical services, public hospitals, and medicines.^17^ People with private health insurance have hospital cover and/or extras cover for non-inpatient healthcare. For women with GBC who need to navigate cancer care services and maternity care services, there may be both public and private health system support required.^18^

The literature provides a large body of evidence exploring the psychological impact on women with breast cancer. Research indicates that women who have experienced GBC face significantly higher levels of psychological distress.^19^ The impact of cancer can create feelings of disconnection from their pregnancy.^20^ These women often have doubts, insecurities, and fears about losing their child, treatment impact on their child’s development, and fears about their own mortality.^19^ A study by Henry et al.^19^ suggests that pregnant women respond with heightened distress when diagnosed with cancer.

The individual risk for breast cancer increases with a family history of the disease, especially when relatives are diagnosed at a younger age.^21^ It is estimated that about 5% to 10% of breast cancers are hereditary.^22^ Genetic risk factors play a relevant role in breast cancer (BC) predisposition, with important implications in prevention and treatment.^21,22^ The BRCA1 and 2 genes, which stand for “BReast CAncer genes”, are the primary genes associated with hereditary breast and ovarian cancer.^22^ However, other genes have also been linked to a breast cancer risk.^22^ Women who inherit variants in the BRCA1 or 2 genes tend to develop cancer at younger ages compared to those who do not carry these genetic variants.^23^ According to Martínez et al., ^24^ breast cancer in women diagnosed with GBC is associated at a rate of 50% with a positive family history and a 30% chance of carrying the mutated breast cancer genes BRCA1/BRAC2.^24^ One-third of women diagnosed with GBC also have a subtype of triple-negative breast cancer (TNBC).^24^ Women diagnosed with GBC may also carry germline mutations contributing to unique psychological, educational, and informational requirements.^25^

Women diagnosed with genetic breast cancer require information that improves their understanding of their disease causes, the specific subtype of breast cancer, and any genetic risk factors that may be involved.^11^ Explaining genetic information and prognosis to patients in clinical terms requires both sensitivity and clarity. As women process this information, they think about their partners, their family, survivorship, and options for fertility.^25^

The findings from a scoping review informed the development of the interview guide designed for this study.^26^ In March 2022, a discussion about the progression of the study for women with GBC and any further advice was gained from Cancer Voices NSW Consumer representatives. After completing the scoping review, the authors designed an exploratory qualitative study to address the research question: “What are women’s experiences with GBC, and how do they describe their interactions with the healthcare system?”

## Methods

### Design

This qualitative, exploratory study aimed to examine the experiences of women with GBC and their interactions with the healthcare system. The design was used to describe the experiences, perceptions, and insights of women diagnosed with GBC. Exploratory research often involves small participant numbers, which allows the researcher to gather first-hand information.^27^ Small participant numbers can provide comprehensive data that enables in-depth analysis. This design is essential when there is little or no information about a particular phenomenon available.^28^ A deeper understanding of the issue can assist in deciding whether further research is necessary.^29,30^

In the study, a qualitative methodology is used to describe and understand the meanings and experiences of the women with GBC as perceived by them.^31^ This study used an interpretive framework to explore women’s experiences, which allows the researcher to explore and identify potential areas of improvement to strengthen and support women with GBC within the healthcare system.^32^ The study is reported in accordance with the consolidated criteria for reporting qualitative research (COREQ): a 32-item checklist for interviews and focus groups.^33^

### Participants and recruitment

Participants were recruited using convenience sampling, which consisted of those who volunteered or self–selected.^34,35^ In qualitative research, inclusion criteria are crucial for ensuring that participants can provide the necessary information to address the research question.^36^ The inclusion criteria for participation in this study were women: aged 18 years or older, able to communicate effectively in English, and diagnosed with GBC within the last 10 years.

The recruitment process involved contacting various nationwide and local breast cancer support groups, community organisations, national cancer websites, volunteer groups, charities, and social media. Snowballing was also used as a recruitment strategy to locate eligible women with GBC. Potential participants contacted the researcher by email or phone to ask any questions or seek clarification before establishing an interview date. The researcher sent participants a Participant Information Sheet (PIS) and a consent information sheet via email (see Supplemental Appendix G, version 2). The PIS outlined the study’s purpose, the consent process, and how the study contributed to the researcher’s PhD. Verbal consent was obtained from the subjects and was audio-recorded.

Open-ended, semi-structured interview questions were employed to gather information. Field notes were made immediately after the interview. A GBC interview guide in Table 1 was prepared based on the research question to facilitate the interview process.

**Table 1.** Interview questions for women with GBC.

The first few questions I will ask the participant to provide demographic data and to break the ice. In what state or territory do you live? In which setting do you reside, that is, metropolitan, regional, or rural? Describe your family and support networks.
1. Tell me about your experience of GBC?
1a. Prompts: age; year of diagnosis; pregnancy
2. Tell me about your treatment?
2a. Prompts: different departments, different clinicians, what worked well, what did not work well, what could be improved.
3. Is there anything else you would like to add?

GBC: gestational breast cancer.

### Data collection

Interviews were undertaken between November 2021 and June 2022 and followed a semi-structured approach. The study was conducted during the COVID-19 pandemic regulations; therefore, interviews were conducted via telephone, audio-recorded, and no repeat interviews were carried out. Confidentiality and anonymity are essential ethical principles in research. During the phone interviews, verbal consent was obtained from participants, ensuring their data privacy. The interviews took place between the researcher and the participant. Following consent, the phone interviews were audio-recorded using a digital recorder and saved as an MP3 file. Interviews ranged from 29 to 67 min in length, with the median duration of 46 min. A professional transcription service transcribed each digital file verbatim into a Word document. The researcher thoroughly verified the accuracy of the transcripts by reading the data transcripts and listening to the digital audio-recording multiple times. Afterward, the transcripts were copied and pasted into an Excel spreadsheet, while additional columns were added for data analysis.

### Data analysis

The data analysis for this study was conducted using the framework of thematic analysis established by Braun and Clarke (2006) six steps.^37^ Thematic analysis is a widely used qualitative research method well-suited to exploratory qualitative research, in which the goal is to uncover and interpret patterns or themes within interview transcripts.^38^

The thematic analysis framework provides a theoretical paradigm, an analytical focus, and guidance on extracting meanings from the interview data. This differs from analysing quantitative data sets as this approach often evaluates data focused on numbers, inferential statistics, cause and effect variables organised by charts and graphs.^38^ Thematic analysis framework guided the researcher in detecting and evaluating relevant patterns in the data. Focusing on the meanings behind the data allows the researcher to describe women’s experiences of GBC.^39^

The six steps involved becoming familiar with the data, integrating codes into themes, reviewing them, determining their significance, and reporting the findings.^37^ The method explores the realities that individuals construct, resulting in textual accounts of their lives and their world.^40^ The method identifies data themes, addresses the research question, and explores the phenomena.^41^

The first step of the analysis involved the researcher reviewing the interview data. Then, the content is read line by line without attempting to rationalise or interpret it. Then the researcher and three supervisory researchers reviewed, repeatedly identified, and analysed the transcripts, interacted with the data, made notes on any words, meanings, insights, and agreed on any preliminary codes. The process was conducted to examine the data thoroughly, including any linkages, connections, and meanings.

The second step in transcribing the data involved creating precise codes next to each column. The third step was to analyse all the codes for sub-themes and themes with greater significance and meaning derived from the data. In the fourth step, some sub-themes were combined and divided into new categories relevant to the study question. The fifth step was to create a central idea for each theme. Finally, the sixth step involved writing a report summarising the viewpoints and experiences of women with GBC. Examples of the analysis process from meaning units to themes are found in Table 2. Alphanumeric coding was employed to de-identify the data, providing adequate data identification and organisation method.

**Table 2.** Examples of the analysis process from meaning units into themes.

Meaning units	Codes	Sub-theme	Theme
I was absolutely devasted, and obviously my main concern was that I wasn’t going to make it, and how was I going to have treatment while I was pregnant, I cried for about five weeks straight, but yeah, that’s how it’s felt. Absolutely devastated. My happiness was just stolen from me (W1).	Absolutely devasted, disbelief, shock, not going to make it, death, confusion around treatment and pregnancy, feel anxious cried for weeks	Absolutely devastated	My happiness was stolen
I think there’s multiple things. There’s worry for your unborn child, obviously, if they’re going to have long‑lasting effects from chemo, if they’re going to be born safely, if you’re going to have a healthy baby at the end of it. How you’re going to cope, how you’re going to cope with your existing child, how you’re going to cope with two children 18 months apart. Not being able to breastfeed and guilt over that. Not being a good parent to your existing child because you’re sick all the time and can’t care for them the way (W4).	Worry about unborn child, questioning any long-lasting effects how to cope with existing child, how to cope with two children, guilt of not being able to breastfeed, not being a good parent, can’t care for them the way they, your sick all the time, wanting to go to dad more	I failed as a mum	
Look, I think one of the things that I kind of always regretted was not having enough information and this is probably specific to pregnancy-related breast cancer, but just breast cancer in general. But I didn’t have enough information going in (W3).	Limited information on GBC, Limited information on breast cancer generally, mastectomy other surgical options, expanders, implants	I didn’t have enough information	It really knocked me around
There was one day I think, where I saw somebody who looked about my age. Everybody else seemed so much older. Like, I’d still chat to them but yeah, I didn’t feel I often saw people similar to me there (W5).	Everyone seemed older, feel different, feel no one was similar	We are not the same	
It was the fact that I couldn’t breastfeed, because I really loved it with my first two, and just the bond that it creates and just that it was being taken away from me. I didn’t have a choice, you know? (W1).	Couldn’t breast Bonding Breastfeeding option taken away I didn’t have a choice	I didn’t have a choice	I wanted control
My breast surgeon was going in there advocating for me, saying, she’s really concerned. This is what I’m thinking of doing. What do you guys think? That’s how they came up with the whole plan about how to treat me. I felt like I was being heard from the doctor’s point of view (W2).	Surgeon advocating for me Treatment plan was about me Felt I was being heard	I felt like I was heard	

GBC: gestational breast cancer.

During this study, our approach to saturation involved reading and re-reading each transcript, identifying codes, and then identifying whether any new aspects, dimensions, or distinctions were identified in subsequent transcripts.^42^ Although the sample number was small, saturation was deemed to have been reached when no new issues or codes had emerged, and subthemes and themes developed sequentially from the transcripts.

### Data quality control

Several approaches were employed to ensure transparent reporting, reliable data collection and analysis. The research advisory team has extensive experience in qualitative research, design, and data analysis. The exploration of results was discussed and checked against the data passages and the codes, subthemes, and themes represented the participants’ ideas, feelings, and perceptions. During the data interpretation phase, the researcher engaged in reflexivity and acknowledged any preconceptions or predispositions that may have impacted the initial interpretation of the data.

Reflexivity is a research practice that involves critically reflecting on one’s personal biases in research.^34^ One’s subjectivity is monitored as a resource for analysis and interpretation in all stages of the research process and theme analysis.^38^ In this study, the researcher had prior experience as a cancer nurse conducting patient interviews. However, conducting interviews with women who had GBC proved to be a different experience. The researcher carefully asked specific questions without steering the conversation, using precise wording and follow-up questions to gain clarity. The researcher thought about these distinctions and stayed watchful to ensure that the interviews were conducted accurately. Orb et al.^43^ state that separating these two roles as a researcher and nurse can be challenging. Reflexivity was practiced, and frequent reviews were conducted with the research team.

## Results

### Characteristics of participants

A total of six women who met the inclusion criteria were interviewed, and none of them declined to participate. Demographic data were collected from the women as presented in Table 3. Four of the women lived in metropolitan areas of major cities, and two lived in regional cities across five Australian states. Five of the women were diagnosed with TNBC, with one also resulting in a BRCA1-positive test. One woman was diagnosed with an excess of human epidermal growth factor 2, a (HER2) positive result.

**Table 3.** Demographic, obstetric, and diagnostic characteristics of participants.

Woman’s code	Geographical location	Age at diagnosis	Breast cancer diagnosis	Gestation at diagnosis	Parity category	Relationship status
W1	Metropolitan Western Australia	39	TNBC BRAC1-positive	32 weeks pregnant	Multiparous	Partner
W2	Metropolitan ACT	41	TNBC	8 weeks post-partum	Primiparous	Single
W3	Metropolitan ACT	33	TNBC	13 weeks pregnant	Multiparous	Partner
W4	Regional New South Wales	33	TNBC	21 weeks pregnant	Multiparous	Partner
W5	Regional Queensland	34	TNBC	12-week post-partum	Primiparous	Partner
W6	Metropolitan Victoria	33	HER2-positive breast cancer	39 weeks pregnant	Primiparous	Partner

TNBC: triple negative breast cancer.

Four women were diagnosed between 13 and 39-weeks’ gestation and two women were diagnosed between 8- and 12-weeks post-partum. Three women received the diagnosis during their first pregnancy, two during their second pregnancy and one during their third.

The qualitative data analysis was conducted iteratively to explore and understand the themes and their meanings. The research advisory team carefully reviewed each step to ensure it was relevant, consistent, accurate, and substantive.

### Themes identified

**Figure 1.**
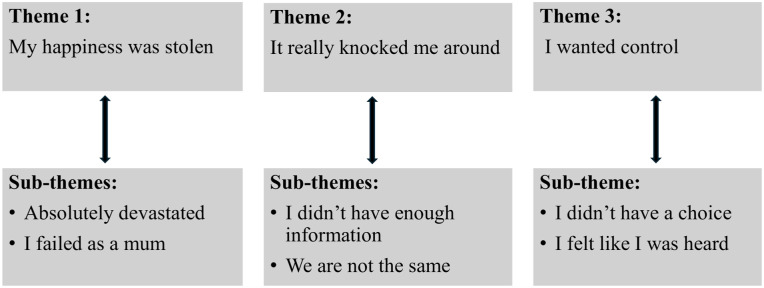
Themes and sub-themes of the experiences of women with GBC. GBC: gestational breast cancer.

In the study, three overarching themes were identified from the analysed data: (1) “*My happiness was stolen*”, (2) “*It really knocked me around*” and (3) “*I wanted control*”. Six sub-themes included “*Absolutely devastated*”, “*I failed as a mum*”, “*I didn’t have enough information*”, “*We are not the same*”, “*I didn’t have a choice*” and “*I felt like I was heard*”. The themes and sub-themes are presented in Figure 1.

### Theme 1: My happiness was stolen

The overarching theme “My happiness was stolen” captures the women’s unique psychological reactions concerning their diagnosis. Several women expressed emotional distress when they realised their world had changed dramatically. Two sub-themes emerged under this theme: “Absolutely devastated” and “I failed as a mum”.

I cried for about five weeks straight, but yeah, that’s how it’s felt. Absolutely devastated. My happiness was just stolen from me (W1).

Several of the women described being “hysterical”, “crying it out”, “I was totally unsuspecting”. “very emotional” and “I was absolutely in shock”. While the women felt overwhelmed, they also acknowledged that they were in the stages of denial while undergoing investigations and tests. These reactions acted as a protective response during stressful situations.

Right up through to the biopsy, which they said needed to be done urgently, I still was thinking it’s not a big deal (W5).

#### Sub-theme: Absolutely devastated

A range of emotional reactions were expressed from discovering something unusual to further investigations, resulting in a worrying period described as being “terrified”, “completely floored”, “concerned for their baby”, “a death sentence”, and the distraught of being diagnosed while also being a new mum. The women reported this experience as being “absolutely the most shocking, upsetting time in my whole life”; for some of the women, this was the first time they had experienced such a serious health issue. This involved one woman “crying for several weeks”, and “being in shock about the whole thing”.

It’s just not something I’d ever heard of happening. Yeah, it was just completely – like I just was completely floored (W6).

I was absolutely devastated, and obviously my main concern was that I wasn’t going to make it, and how was I going to have treatment while I was pregnant (W1).

#### Sub-theme: I’d failed as a mum

During this time of uncertainty, the women reflected on their experiences of motherhood and feelings of guilt and failure at not being able to provide adequately for their child/ren. One woman shared her feelings, “I am such a terrible mother”, and another woman expressed fear of “not being a good parent” as being unable to care for her child, usually while undergoing treatment. This sub-theme emphasises the importance of loss, identity, femininity, body image, lactation, and the transition to motherhood in relation to how women see themselves as mothers.

There were a lot of tears, a lot of me telling my husband that I’d failed as a mum and that I was such a terrible mother (W3).

Not being a good parent to your existing child because you’re sick all the time and can’t care for them the way that they. . . (W4).

### Theme 2: It really knocked me around

Breast cancer treatments during pregnancy can be a challenging experience for women and their families. The treatment options for GBC include chemotherapy, radiotherapy, surgery, hormone therapy, and breast reconstruction. This theme was directly linked to the women’s experiences with medical treatments. One woman stated that “as soon as baby was born, I was straight into all the machines”. For other women, some of difficulties related to commencing treatment as expressed by the women: “I just finished breastfeeding”, and “after the bone scan, I was unable to hold my baby”, “a seroma after a lumpectomy”, “my egg collection got put on hold”, “first time spent away from my baby”, were pivotal challenging moments.

The women reported difficulties handling treatment, one stated, “I would have zero neutrophils, treatment was often delayed”, “after chemo a double mastectomy”, “I went into surgery crying”. They also shared their concerns about the impact on their fear of treatment, and its impact on child/ren, family and future pregnancies.

. . .I just was so mentally knocked. Like I just was – I couldn’t – I wasn’t in the right headspace anymore, so I decided to have a caesarean (W6).

I was really sick. I felt really sick for about seven or eight days after the treatment (W2).

Many women shared their experiences regarding the physical effects of treatments received for GBC and diagnostic tests. The effects ranged from common symptoms such as “having chemo brain”, “ended up with gestational diabetes”, “I was really, really sick”, “sitting in emergency in a bed, shivering, I’m feeling absolutely awful”, to more severe and unexpected reactions such as allergic reactions during treatment, being unprepared for painful procedures, invasive tests, and feeling physically exhausted. Some women also expressed feeling overwhelmed and unprepared for these side effects.

To be honest, when I was going through chemo, chemo really knocked me around. My levels were completely dead. It was tough (W2).

#### Sub-theme: I didn’t have enough information

Several women in the study emphasised the significance of effective communication between doctors, nurses, and midwives during their pregnancy, diagnosis, and treatment of GBC. Establishing trust, receiving guidance, and setting clear objectives are critical for effective communication. While most women expressed that communication was well received, informed, and prepared. There were situations where information and communication between doctors, nurses, and midwives was unclear, indirect, and difficult to understand.

. . .when you get that diagnosis of, you’ve got cancer, knowing where to go or knowing what questions or who to ask, is probably one of the biggest things that could be improved . . .(W2).

The women highlighted the importance of being well informed, open, and time to prepare for discussions.

Look, I think one of the things that I kind of always regretted was not having enough information, and this is probably specific to pregnancy related breast cancer, but just breast cancer in general. But I didn’t have enough information going in, like when they told me I needed to have a mastectomy I wasn’t given the option of having an expander put in or anything (W3).

#### Sub-theme: We are not the same

This study identified that the women underwent a broad range of feelings, such as sensing a reason for their uniqueness, lacking priority in care, being a woman, feeling different from other cancer patients, standing out, having distinct responsibilities during treatment, such as caring for a newborn, and having different needs when it comes to self-care and raising a family.

One, explained:

. . .we don’t treat you any different because you’re pregnant, and I was like, well, I wasn’t happy with that, because it is different being pregnant . . .. (W1).

While another stated:

. . .I think I was telling myself that they don’t have women in my position come through very often . . .(W5).

The women in the study highlighted that HCPs seemed unaware of these differences. The differences and challenges of childbirth, pregnancy, and motherhood contributed to their sense of being different.

. . .sometimes I think they forget about the human element in it and maybe some of the challenges you’re experiencing in terms of understanding that I have a toddler at home that I’m still caring for, plus I’m going through this, plus I’m heavily pregnant. Often, they would forget that I just can’t go home and rest . . . (W4).

There was one day I think, where I saw somebody who looked about my age. Everybody else seemed so much older. Like, I’d still chat to them but yeah, I didn’t feel I often saw people similar to me there (W5).

### Theme 3: I wanted control

The women in the study expressed several reactions to the diagnosis of GBC, and one that stood out was being in control. The women raised the importance of feeling in control and taking charge in certain situations, and when situations dictated that they had no control. The women rated their ability to influence certain situations and their surroundings as essential to feeling in control.

One woman stated:

Having the space to do some research and talk to my doctor about it, I decided that I really wanted to have a preventative - I wanted to have a full mastectomy on the cancer side and a preventative mastectomy on the other side, so a full double mastectomy with immediate reconstruction. So, I sought out through my doctor another surgeon who does that and switched over to her. In one way, I was able to make my own decision in terms of surgery options (W4).

Understanding cancer treatment decisions can be challenging to make sense of, and participating actively in decision-making was crucial to their physical and mental well-being.

My body knew what it was doing more the second time, I guess, as it does. I probably was very mentally determined that I wanted control. That was my one thing that I felt like I had control over, so my mindset was very much, I can do this (W4).

. . .I ended up choosing to have the other side done at the same time, so I mean that obviously made it a bigger surgery (W3).

The women also expressed their confidence in asking questions and advocating for themselves. The women described how their relationships with HCPs played a pivotal role in helping them feel comfortable asking questions, advocating for themselves, and making decisions.

One woman stated:

I’m pretty good at advocating for myself and making sure I get good information. It’s not something that was brought up unless I did . . . (W2).

While another said:

I ended up questioning the surgery that I needed to have, and I ended up changing surgeons to a surgeon that I felt more comfortable with (W4).

#### Sub-theme: I didn’t have a choice

The women involved in this study were confronted with the complexities of considering sensitive medical and surgical treatment decisions related to their pregnancies, what was meant by a diagnosis of GBC, and considerations regarding the delivery or caring for their child/ren.

One woman stated:

so, she wanted to start treatment straight away, and I just felt that she wasn’t listening when I said I wanted to wait because of the baby (W1).

They had to carefully consider the implications of these decisions for themselves and their child/ren. Although none of the women in this study terminated their pregnancies, they reported feeling fear of this situation but also confounded by external factors that limited their choices, expressing a need for time to process their situations. These women often faced time challenges such as the urgency of treatment, decisions relating to breastfeeding, and conflicting expectations that did not always align with their personal values and goals.

One woman stated:

. . .Oh gosh, honestly, it was just so upsetting. All my decisions were pretty much being made for me . . . So, just being out of control, which is really hard. . . (W1).

It was very hard, very hard for me. I just got on with it because you have no other choice. I pushed it out of my mind because I was, like, I have no other option to do it, but it was a bittersweet moment for me (W4).

#### Sub-theme: I felt like I was heard

In this study, the women actively participated in their care during the treatment of GBC. This involvement included discussing their fears and concerns with their doctors, nurses, and midwives, their desires to breastfeed, surgical options, having more children, fertility preservation, and worries about their babies during treatment.

One woman reported:

. . .so they knew that I wanted to breastfeed, so they timed my chemo to finish three weeks before I was due and then they induced me on that date . . . (W3).

The women also took the initiative to research information about surgical incision options and breast reconstruction.

One woman stated:

Yeah so, went to the surgeon with this huge like case - business case of why I need a double mastectomy, and I was like just straight up I said look I think I want a double mastectomy, and she said I think that would be a good idea. I was like, oh (W5).

The women often presented their findings to their doctors or surgeons, and their requests were

often considered and integrated into their treatment plans.

. . .I said, this is my concern. What are my options? . . . my breast surgeon was going in there advocating for me. That’s how they came up with the whole plan about how to treat me. I felt like I was being heard from the doctor’s point of view . . . (W2).

## Discussion

This study explored the experiences of six women diagnosed with GBC and their interactions with healthcare systems in Australia. Three main themes emerged from the collected data: “My happiness was stolen”, “It knocked me around”, and “I wanted control”. The first theme highlights that the women faced profound psychological challenges when confronted with a pregnancy or post-partum breast cancer diagnosis.

The women in this study experienced unique psychological reactions concerning their diagnosis of GBC, such as devastation and uncertainty in relation to their motherhood and future family prospects. These findings are akin to those of previous qualitative studies.^19,44–46^ Faccio et al.^47^ reported that women with GBC face more complex psychological challenges connected to the transition to motherhood. Likewise, Ives et al.^46^ state that the decisions made by a woman with GBC are unique to her individual circumstances, her views on motherhood, and her desire for more children all play a major role in the psychological impact on women.

Significant psychological distress related to their diagnosis was experienced by the women in this study, and several of them sought out additional psychological support services to address their emotional well-being. The women in this study reported that the reasons for seeking additional psychological supports was because the services offered primarily focused on counselling related to a cancer diagnosis, while counselling addressing pregnancy or post-partum and breast cancer was limited. These findings align with Stafford et al.’s ^14^ qualitative study, which indicated that women with GBC had additional mental health and supportive care needs that were met to varying extents. This experience is consistent with Rees et al.’s^48^ study, which noted that although counselling may be beneficial, further research is necessary to fully understand the psychological impact on women with GBC. In addition, these findings are supported by Facchin et al.^10^ study which argued that the psychological effects experienced by women with GBC are often overlooked in the context of women’s health.

Women diagnosed with GBC in this study reported the diagnosis significantly impacted their emotional well-being, experiences with cancer, motherhood, and pregnancies. For some of the women, this was their first encounter with a serious health issue, raising concerns to HCPs about wanting another child, fertility, and breastfeeding being difficult. It was important for the women to feel reassured by the HCPs. These findings are supported by Faccio et al.^47^ which emphasised that the relationship between the mother and the baby begins during pregnancy. These findings are similar to a case study by Zanetti-Dallenbach et al.^45^ where the authors emphasised the importance of women with GBC feeling that all medical professionals are focused on the well-being of both the mother and baby as a whole.^45^

In this study, the women expressed distressing emotions and extreme anxiety. These reactions were found in a quantitative study by Henry et al.^19^ where the authors showed that women with GBC experienced a higher risk of long-term psychological distress as measured by the Impact Event Scale. Their study found that up to 51.5% of women with GBC reported clinical distress, compared to 33% of women with breast cancer.^19^ Recognising the early signs of psychological distress in women with GBC is essential, and timely referrals to psychosocial services are necessary to ensure these women receive appropriate support.^19^ However, according to Stafford et al.^14^ accessing these supports can be challenging.

The second theme, “It really knocked me around,” pertains to the women in this study experiencing physical complexities and side effects from treatments and procedures for GBC. The women in this study experienced an urgency for diagnostic tests, procedures, and treatments such as procedures for lines and devices for administration of chemotherapy, bone scans, and surgery. These experiences physically and emotionally impacted the women, which is supported by Farhana et al.^49^ as many women felt a sense of urgency or pressure immediately after the diagnosis of GBC. Another study by Liow et al.^50^ found that the women expressed an altered appearance from treatments, causing distress and affecting their self-esteem.

The women in the study experienced the first time being away from their baby and the first time experiencing major interventions such as surgery, chemotherapy, radiotherapy, and a caesarean birth. Similar to Rodsten,^51^ the women described how they felt themselves thrown into a sudden psychological and physical dilemma involving their own survival and the survival of their unborn child. The women in this study shared their struggles in coping with the toxicities of chemotherapy, side effects of endocrine treatment, and physical challenges from surgeries. In contrast, the women in Hammarberg et al.’s^44^ study reported being less concerned about the physical effects of treatments on their own body and how it might affect their baby; however, they were concerned about short- and long-term effects such as lymphoedema, weight gain, and menopause.

While the women in this study reported feeling extremely unwell, needing hospital admission, all while caring for a newborn, they also felt different from other women with breast cancer. These findings are shared by Facchin et al.^10^ with their participants experiencing a sense of difference related to physical changes and psychological sufferance. Similar findings were reported in Ives’s^52^ mixed-method study and Ferrari et al.’s^53^ research. Participants in both studies expressed feelings of being different from other mothers while experiencing isolation because they struggled to identify with any other women who had undergone similar circumstances.

The women in this study expressed that there were instances during the diagnosis and treatment phases of breast cancer where they required customised information and communication regarding the risks and benefits. This included information on fertility treatments, the implications of pregnancy-related breast cancer, cancer treatment options, and surgical alternatives. The importance of women being well informed and prepared during a diagnosis of gestational cancer is supported by Farhana et al.^49^ as many of the women felt ill prepared regarding the situation, and HCPs also lacked adequate knowledge. In addition, in Stafford et al.’s^54^ study, the women reported seeking tailored information about their condition and treatments as well as one-on-one peer support with other gestational cancer survivors, which was challenging for them to access, and clinical services struggled to provide.

The third theme, “I wanted control”, focuses on the complexities of decision-making by the women in this study. The women emphasised the significance of their contributions and values in decision-making, as well as the need for a sense of control. The women in this study highlighted the importance of taking charge of their circumstances whenever possible. Similarly, Stafford et al.^14^ found women reported a sense of control in treatment scheduling, the importance of trust in HCPs, coordination of care, and good communication were positive experiences.

The ability for women with GBC to make their own decisions and have opinions, options, and choices greatly affected their psychological well-being. These findings align with research by Kozu et al.^55^ and Vandenbroucke et al.,^56^ which indicate that the decision-making process for pregnant women with cancer is both complex and critical to their overall physical and psychological health. Furthermore, Rocque et al.^57^ shared that decision-making is a fundamental principle of high-quality healthcare. Patients value being informed of the risks and benefits associated with treatment options that reflect their preferences and values.^57^ This highlights the importance of integrating woman-centred care, a point that resonates with these study findings, where advocacy and decision-making significantly contribute to the women’s psychological health and well-being.

In contrast, some of the women in this study often felt pressured, that decisions were made for them by HCPs, they were not heard, and at times were not given a choice during the diagnosis, treatment, and management of GBC. However, other women reported they felt very supported by their clinical team, who listened to and felt extremely comfortable with the decisions being made. This study’s findings are supported by Gomes et al.^20^ that women with GBC decisions and actions depended on how they defined the current situation, on their interactions with HCPs, and on their thought process in determining their best action.^20^

The women in the study reported that they felt most challenged in making decisions about breastfeeding, fertility preservation, reproduction, breast reconstruction, and surgery types. Several studies emphasise that effective communication and thorough explanations can significantly benefit women to take a more active role in these decisions.^19,44,45^ In addition, these findings align with Stafford et al.^14^ stating shared decision-making is associated with increased perception of patient control and HCP satisfaction. Whereas a study by Ussher et al.^58^ stated that while HCPs were aware of the importance of decision-making and providing fertility options for women with cancer, they reported that they only addressed this issue 50% of the time, and 24% of the time for allied health. This outlines the importance of the relationship and communication between the HCPs and the women being empowered and actively involved in their care.

### Strengths and limitations of the study

The research utilised a qualitative exploratory design to examine women’s experiences with GBC and their interactions with healthcare services. Although the sample size was small, it is an appropriate sample size for this methodology and provided valuable data to generate important insights into an unexplored area, the experiences of women with GBC and their interactions in the Australian health system.

Convenience sampling was used, and this has inherent limitations. The women who elected to participate may be relatively homogeneous and not diverse, and therefore biased.^35,59^ The study was also limited by its focus on participants from metropolitan and regional areas; the experiences of women in rural and remote areas with GBC could have presented different experiences.

### Recommendation for further research

Future research into the experiences of women with GBC should focus on the specific concerns highlighted by the women in this study. This includes investigating the psychological, social, and peer support services available to these women and assessing the effectiveness of these services in helping them cope with both breast cancer and pregnancy. In addition, more research is needed to examine the physical effects and impacts of the different treatments on women with GBC. These inquiries could encompass various aspects such as experiences post-surgery, decisions regarding breast reconstruction, side effects from treatments that resulted in hospitalisation, treatment effects on fertility, in vitro fertilisation, embryo preservation, and the management of early menopause. Exploring these questions could lead to improved preparation and care for women undergoing treatment and management for GBC.

Further research is needed on tailored information and resources to support both HCPs and women with GBC in their discussions and decision-making processes, helping to identify their needs and prepare for adequate support.

### Implications for policy and practice

The findings highlight the importance of healthcare services improving their policies and practices to provide the most effective psychological care and support for women experiencing GBC. Healthcare organisations should adopt a woman-centred approach that includes effective communication strategies and customised information about the treatment and management of GBC. This will assist women in making informed decisions about their well-being and the well-being of their unborn child.

## Conclusion

This study examined the complex challenges faced by women diagnosed with GBC, highlighting a difficult situation causing increased distress due to the combined pressures of being pregnant while undergoing chemotherapy, surgery, and radiotherapy, as well as caring for a newborn or other children, and their desire to have more children in the future.

These findings offer valuable opportunities for doctors, nurses, and midwives to enhance their approach to prioritising the psychological well-being of women with GBC. It is essential to emphasise effective communication and actively involve these women in the decision-making process. This includes considering their preferences, providing tailored and relevant information, and clearly explaining the cancer diagnosis and prognosis. Sensitivity and clarity throughout the care process are crucial in supporting women with GBC.

## Supplemental Material

sj-docx-1-whe-10.1177_17455057261435753 – Supplemental material for What are women’s experiences of gestational breast cancer, and how do they describe their interactions with the healthcare system? An exploratory study Supplemental material, sj-docx-1-whe-10.1177_17455057261435753 for What are women’s experiences of gestational breast cancer, and how do they describe their interactions with the healthcare system? An exploratory study by Sara Hurren, Marie McAuliffe, Karen Yates, Tracey Ahern and Cate Nagle in Women's Health
