# Supplementary material for: What are women’s experiences of gestational breast cancer, and how do they describe their interactions with the healthcare system? An exploratory study
Source: Womens Health (Lond). 2026 Mar 29;22:17455057261435753. doi: 10.1177/17455057261435753 (PMC13033891; doi:10.1177/17455057261435753)
Supplement: sj-docx-1-whe-10.1177_17455057261435753 – Supplemental material for What are women’s experiences of gestational breast cancer, and how do they describe their interactions with the healthcare system? An exploratory study [file sj-docx-1-whe-10.1177_17455057261435753.docx]

### APPENDIX G

### PARTICIPATION INFORMATION SHEET

What is the experience of women with gestational breast cancer and their interactions with the health care system?

If you have experienced pregnancy and breast cancer within the last ten years, are aged 18 years or older and able to communicate in English, you are invited to participated in this study.

This study aims to contribute to an understanding of the experiences of women with gestational breast cancer (GBC) and their interactions with the health care system. For this study, GBC is defined as a breast cancer diagnosis during pregnancy or in the first twelve months after the completion of pregnancy. The experiences of women with GBC will provide valuable insights into exploring potential knowledge and service gaps. Understanding the experiences of women will deepen our knowledge between women with GBC and contemporary health care to inform clinical practice and education. Sara Hurren, a nurse, is conducting the study and this will contribute to the qualification of a PhD in Health at James Cook University

.

Participation involves one interview conducted via a videoconference or teleconference and will take approximately 45 minutes. Sara will arrange a date and time that is suitable to you, and with your consent, the interview will be recorded on a digital recorder. Questions in the interview will relate to your experience of pregnancy and breast cancer and aspects of your care within the health care system. Data collection will also include what state you live in and whether you live in a rural, metropolitan, or regional area.

While the questions are general, they may recall instances that may make you feel uncomfortable, and you can pause or stop taking part in the study at any time without explanation. Should you require them, psychological support options include Beyond Blue Australia https://www.beyondblue.org.au, contact number 1300 22 4636; The Cancer Council Australia https://www.cancer.org.au,contact number 131120; Lifeline Australia https://www.lifeline.org.au, contact number 131114.

Your information will be kept strictly confidential by the research team. The data from the study will be used in research, publications, and reports, and you will not be identified in any publication. If you would like a one-page summary of the research findings, please let Sara know at interview or using the contact details below.

If you know of other women who have experienced pregnancy and breast cancer that might be interested in this study, please pass on this information sheet to them so they may contact me to participate in the study. Taking part in this study is entirely voluntary.

If you have any questions, please contact – Sara Hurren.

***Principal Investigator: Primary Advisor***

***Sara Hurren Name: Dr Karen Yates***

***College: Healthcare Sciences College: HealthCare Sciences***

***James Cook University James Cook University***

***Email:*** [***Sara.Hurren@my.jcu.edu.au***](mailto:Sara.Hurren@my.jcu.edu.au) ***Email:*** [***karen.yates@jcu.edu.au***](mailto:karen.yates@jcu.edu.au)

***Phone: 0742321488***

***If you have any concerns regarding the ethical conduct of the study, please contact:***

***Human Ethics, Research Office***

***James Cook University, Townsville, Qld, 4811***

***Phone: (07) 4781 5011 (***[***ethics@jcu.edu.au***](mailto:ethics@jcu.edu.au)***)***

**INFORMED CONSENT FOR INTERVIEWS with women with Gestational Breast Cancer**

The following text will be read to the participant by the PI before the commencement of the interview and audio recorded.

I understand the study aims to explore the women’s experiences of gestational breast cancer and their interactions with the health care system. I consent to participate in this project, the details of which have been explained to me, and I have been provided with a written information sheet to keep.

I understand that my participation will involve an interview conducted by videoconference or teleconference and that this interview will be audiotaped. I agree that the researcher may use the results as described in the information sheet.

I acknowledge that:

- any risks and possible effects of participating in the study have been explained to my satisfaction
- taking part in this study is voluntary, and I am aware that I can pause or stop taking part in it at any time without explanation and withdraw any unprocessed data I have provided
- Any information that I have provided will be strictly confidential, and no names will be used to identify me

______________________________________________________ (please tick to indicate consent)

I consent to be interviewed Yes No

__________________________________________________________________________________

I consent for the interview to be audiotaped Yes No

__________________________________________________________________________________
